# Portal vein surgical treatment on non-tumoral portal vein thrombosis in liver transplantation: Systematic Review and Meta-Analysis

**DOI:** 10.6061/clinics/2021/e2184

**Published:** 2021-01-18

**Authors:** Lucas S. Nacif, Leonardo Y. Zanini, Rafael S. Pinheiro, Daniel R. Waisberg, Vinicius Rocha-Santos, Wellington Andraus, Flair J. Carrilho, Luiz Carneiro-D'Albuquerque

**Affiliations:** I Divisao de Transplante de Figado e Orgaos do Aparelho Digestivo, Departamento de Gastroenterologia, Hospital das Clinicas HCFMUSP, Faculdade de Medicina, Universidade de Sao Paulo, Sao Paulo, SP, BR; II Divisao de Gastroenterologia e Hepatologia Clinica, Departamento de Gastroenterologia, Hospital das Clinicas HCFMUSP, Faculdade de Medicina, Universidade de Sao Paulo, Sao Paulo, SP, BR

**Keywords:** Liver Transplantation, Portal Vein Thrombosis, Systematic Review, Portal Vein, Cirrhotic, Liver Disease

## Abstract

Non-tumoral portal vein thrombosis (PVT) is associated with higher morbidity and mortality in liver transplantation (LT). In this study, we aimed to evaluate the impact of PVT in LT outcomes and analyze the types of surgical techniques used for dealing with PVT during LT.

A systematic review was conducted in Cochrane, MEDLINE, and EMBASE databases, selecting articles from January 1990 to December 2019. The MESH-terms used were (“Portal Vein”[Mesh] AND “Thrombosis”[Mesh] NOT “Neoplasms”[Mesh]) AND (“Liver Transplantation”[Mesh]). The Preferred Reporting Items for Systematic Reviews and Meta-Analysis (PRISMA) recommendation was used, and meta-analysis was performed with Review Manager Version 5.3 software.

A total of 1,638 articles were initially found: 488 in PubMed, 289 in Cochrane Library, and 861 in EMBASE, from which 27 were eventually selected for the meta-analysis. Surgery time of LT in patients with PVT was longer than in patients without LT (*p*<0.0001). Intraoperative red blood cell (*p*<0.00001), fresh frozen plasma (*p*=0.01), and platelets (*p*=0.03) transfusions during LT were higher in patients with PVT. One-year (odds ratio [OR] 1.17; *p*=0.002) and 5-year (OR 1.12; *p*=0.01) patient survival after LT was worse in the PVT group. Total occlusive PVT presented higher mortality (OR 3.70; *p*=0.00009) and rethrombosis rates (OR 3.47 [1.18-10.21]; *p*=0.02)**.** PVT Yerdel III/IV classification exhibited worse 1-year [2.04 (1.21-3.42); *p*=0.007] and 5-year [0.98 (0.59-1.62); *p*=0.93] patient survival. Thrombectomy with primary anastomosis was associated with better outcomes.

LT in patients with non-tumoral PVT demands more surgical time, needs more intraoperative transfusion, and presents worse 1- and 5-year patient survival. Total occlusive PVT and Yerdel III/IV PVT classification were associated with higher mortality. (PROSPERO, registration number: CRD42020132915).

## INTRODUCTION

Non-tumoral portal vein thrombosis (PVT) is a relevant condition in liver cirrhosis evolution, with an estimated incidence rate of 0.7 per 100,000 and prevalence ranging from 0.6-28% in cirrhotic patients (1-3). It is well-established that patients with severe cirrhosis (Child-Pugh C Classification) have a high incidence of PVT (4).

PVT is defined as partial or complete obstruction of blood flow that occurs secondary to a thrombus in the portal vein, which results in the occlusion of the vessel lumen (1-3). Despite this, the decreased portal flow velocity and the increased flow volume are considered independent risk factors in developing PVT (4). There are many classifications regarding the extent of involvement of the portal venous system, with the Yerdel classification being the most used one (5). It is paramount to promptly diagnose PVT, as this may lead to better outcomes and survival.

In the past, PVT in cirrhotic patients was considered a contraindication for liver transplantation (LT). Nevertheless, with the development of better diagnostic tools, medical therapy, and surgical techniques in the last decades, LT became a feasible therapeutic option even for patients with PVT (6). Based on the grade of PVT and severity of the cirrhosis, the decision to perform LT in these patients remains controversial.

Despite the modern advances in with the management of PVT, the outcome of LT in patients with PVT is still under debate, especially in the current era of organ shortage associated with a high model of end-stage liver disease (MELD) score of patients. The main objective of this study was to compare the outcomes of LT in patients with PVT and those without, focusing on the types of PVT surgical treatment, which include thrombectomy with primary anastomosis, interposition vein graft (physiological reconstruction), superior mesenteric vein (SMV) jump graft, interposition vein graft from a collateral vein (non-physiological reconstruction), renoportal anastomosis and cavoportal hemitransposition (CPH).

The present data may help validate whether the different types of portal vein (PV) reconstructions influence transplant outcomes in terms of survival and complications.

## MATERIAL AND METHODS

### Study identification and selection

A systematic review of the literature was performed for intraoperative management of PVT during LT. The Cochrane Library, EMBASE, and MEDLINE-PubMed databases were electronically searched from 1990 to December 2019. The MESH-terms used were (“Portal Vein”[Mesh] AND “Thrombosis”[Mesh] NOT “Neoplasms”[Mesh]) AND (“Liver Transplantation[Mesh]).

The terms and MESH-terms for PubMed database search were developed based on the PICO (patient, intervention, comparison, or control, outcome) structure. The results of the search terms forming the “P” (patients) group were merged with the results of the “I” (intervention) group with an “AND.” To exclude terms, they were merged with “NOT.”

The Preferred Reporting Items for Systematic Reviews and Meta-Analysis (PRISMA) checklist were followed throughout this study (7,8).

Two independent researchers (LSN and LYZ) evaluated the quality and selection of the studies. In the case of disagreement, the researchers held a consensus meeting to reach a final decision.

### Inclusion and exclusion criteria

Within the research question of the PICO structure, the comparison of patients with non-tumoral PVT who underwent LT was defined as inclusion criteria. Only randomized controlled trials, nonrandomized controlled trials, or comparative clinical studies were included. All studies evaluated were written in English. Case series with less than six patients and studies reporting on tumoral thrombosis or Budd-Chiari syndrome were excluded.

### Data synthesis and statistical analysis

Data were extracted from text, tables, and figures of the original published articles. The measures of effectiveness for each treatment were expressed in absolute numbers and respective frequencies, *i.e.*, the absolute risk. For the meta-analysis, the data were synthesized using Review Manager Version 5.4 software provided by the Cochrane Collaboration (RevMan; The Cochrane Collaboration, The Nordic Cochrane Centre, Copenhagen, Denmark). The results from the included papers were compared with the differences observed in absolute risks. Continuous data were expressed as mean difference and 95% confidence intervals (CI).

### Heterogeneity and sensitivity analysis in the studies

Heterogeneity was evaluated with I^2^ statistics, in which I^2^ values of 70% or more represented an indicator of substantial heterogeneity. In the absence of this heterogeneity, we pooled data with a fixed-effect model (I^2^<50%); otherwise, we used a random-effects model (I^2^>50%). Results were considered statistically significant at *p*<0.05.

### Data analysis and critical evaluation

Study quality assessment included study design, level of evidence, and the New Castle score (Ottawa Quality Assessment Cohort Studies) (accessed July 2019) for nonrandomized clinical trials (9).

## RESULTS

### Study selection

**Figure 1:**
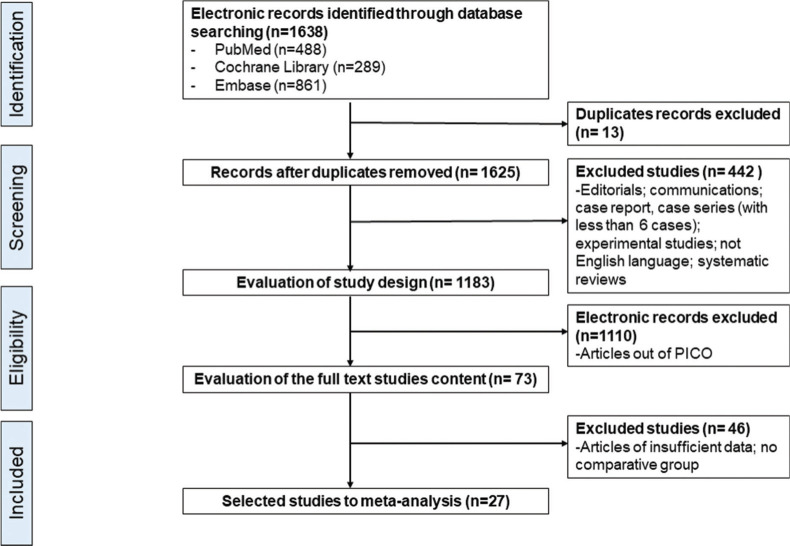
Flow chart of eligible studies selection according to the PRISMA statement.

Using the search strategy mentioned above, we identified 1688 articles, of which 488 articles were from PubMed/MEDLINE, 861 from EMBASE, and 289 from Cochrane Library. After applying the previously defined inclusion and exclusion criteria, 73 articles were selected in the PICO structure, and 27 were eventually included in the meta-analysis, as shown in Figure 1.

The New Castle-Ottawa Scale (NOS) qualification of the studies for non-tumoral PVT in LT and the overall demographics data of selected studies are depicted in Tables 1 and 2, respectively. Table 2 also shows the extent of thrombosis in the portal system according to the Yerdel classification and the types of surgical techniques employed. Thrombectomy was performed in 932 patients, SMV jump graft in 95, interposition vein graft in 23, collateral anastomosis in 32, renoportal anastomosis in 27, and CPH in 50 (Table 2).

### Meta-analysis

For the meta-analysis, 27 articles were selected (5,10-35). Different articles were used for specific data analysis, as shown below:

- Surgery time-7 articles (10,13,20-24);
- Intraoperative transfusion of blood-borne products-8 articles (10,13,21,22,24-27);
- Intensive care unit (ICU) and hospital length of stay-5 articles (10,13,21,26,29)
- Overall survival comparison between PVT and non-PVT patients-19 articles for 1-year survival (5,10-12,14-17,22-25,29-35) and 11 articles for 5-year survival (5,14,16,18,19,22-24,29,32,35);
- Survival comparison between partial and complete PVT-4 articles (5,12,20,24)
- Rethrombosis after LT-6 articles (5,10-12,20,34)
- Survival according to Yerdel classification-3 articles (5,23,24) for 1-year and 5-year survival.

#### Surgery time of LT

**Figure 2:**
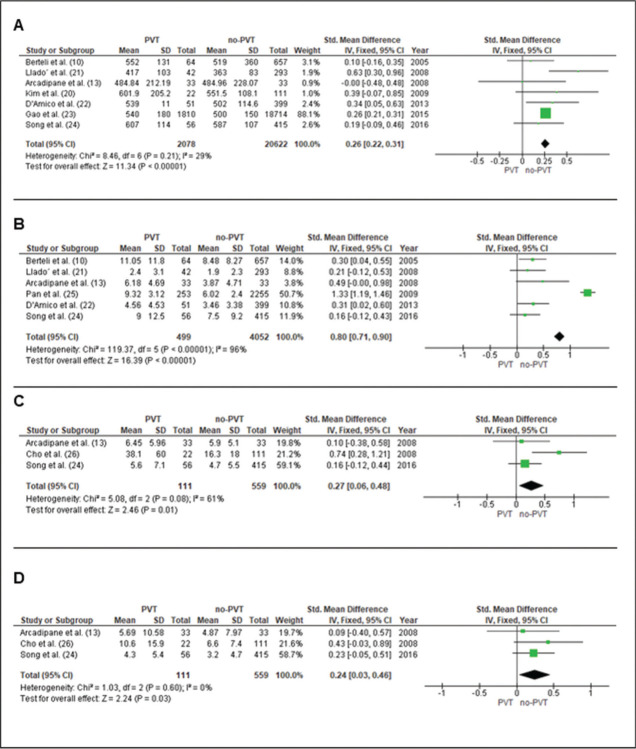
Forrest plot in liver transplantation with non-tumoral portal vein thrombosis (PVT). **A**. Surgery time. **B**. Red blood cells. **C**. Fresh frozen plasma. **D**. Platelets.

Data from seven studies (10,13,20-24) evaluated a total of 22,700 patients (2078 with PVT and 20,622 without PVT). The weighted mean difference was 0.26 minutes [0.22-0.31], *p*<0.0001 (Figure 2A).

### Intraoperative transfusion of blood-borne products

#### Red blood cells (RBC)

Data from six studies (10,13,22,24,25,36) evaluated a total of 4,551 patients (499 with PVT and 4052 without PVT). The mean difference was 0.80 packs [0.61-0.90], *p*<0.0001 (Figure 2B).

#### Fresh frozen plasma (FFP)

Data from three studies (13,24,31) evaluated 111 patients with PVT and 559 without PVT. The mean difference was 0.27 packs [0.6-0.48], *p*=0.01 (Figure 2C).

#### Platelets

Data from three studies (13,24,26) evaluated 111 patients with PVT and 559 without PVT. The mean difference was 0.24 packs [0.03-0.46], *p*=0.03 (Figure 2D).

### Length of stay

#### Hospital length of stay

**Figure 3:**
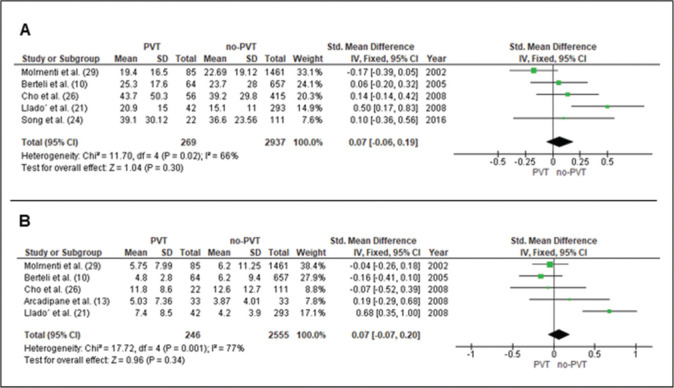
Forrest plots in liver transplantation with non-tumoral portal vein thrombosis (PVT). **A**. Hospital length of stay. **B**. ICU length of stay.

Data from five studies (10,13,21,26,29) evaluated 269 patients with PVT and 2937 without PVT. The mean difference was 0.07 days [-0.06-0.19], (*p*=0.30) (Figure 3A).

#### ICU length of stay

Data from five studies (10,13,21,26,29) evaluated 246 patients with PVT and 2,555 without PVT. The mean difference was 0.07 days [-0.06-0.20], (*p*=0.34) (Figure 3B).

### Survival analyses

#### 1-year Patient Survival

**Figure 4:**
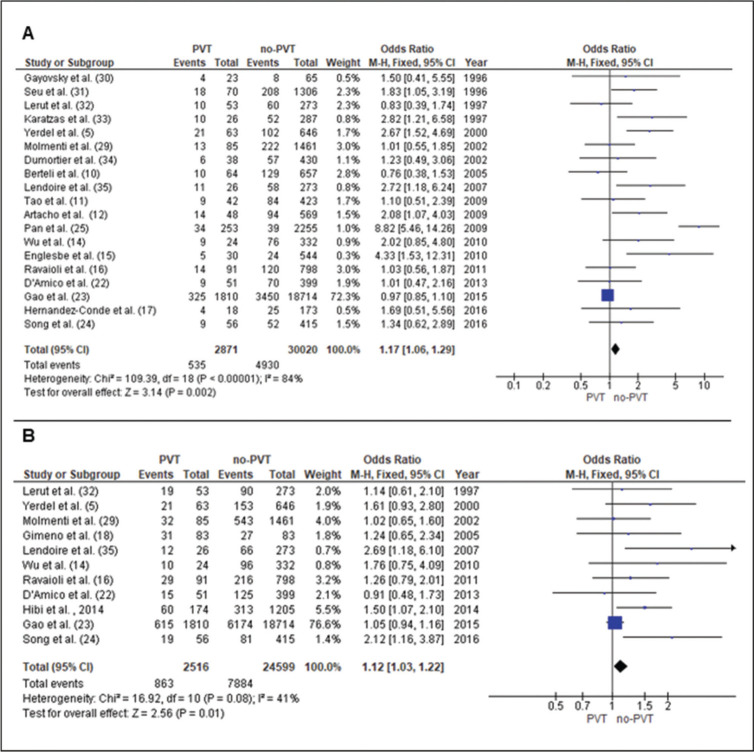
Forrest plots in liver transplantation with non-tumoral portal vein thrombosis (PVT). **A**. 1-year survival. **B.** 5-year survival.

Data from 19 studies (5,10-12,14-17,22-25,29-35) evaluated 2871 patients with PVT and 30,020 patients without PVT. The odds ratio (OR) was 1.17 [1.06-1.29], *p*=0.002 (Figure 4A).

#### 5-year Patient Survival

Data from 11 studies (5,14,16,18,19,22-24,29,32,35) evaluated 2516 patients with PVT and 24,599 patients without PVT. The OR was 1.12 [1.03-1.22], *p*=0.01 (Figure 4B).

#### Partial *vs.* total PVT 1-year Patient Survival

**Figure 5:**
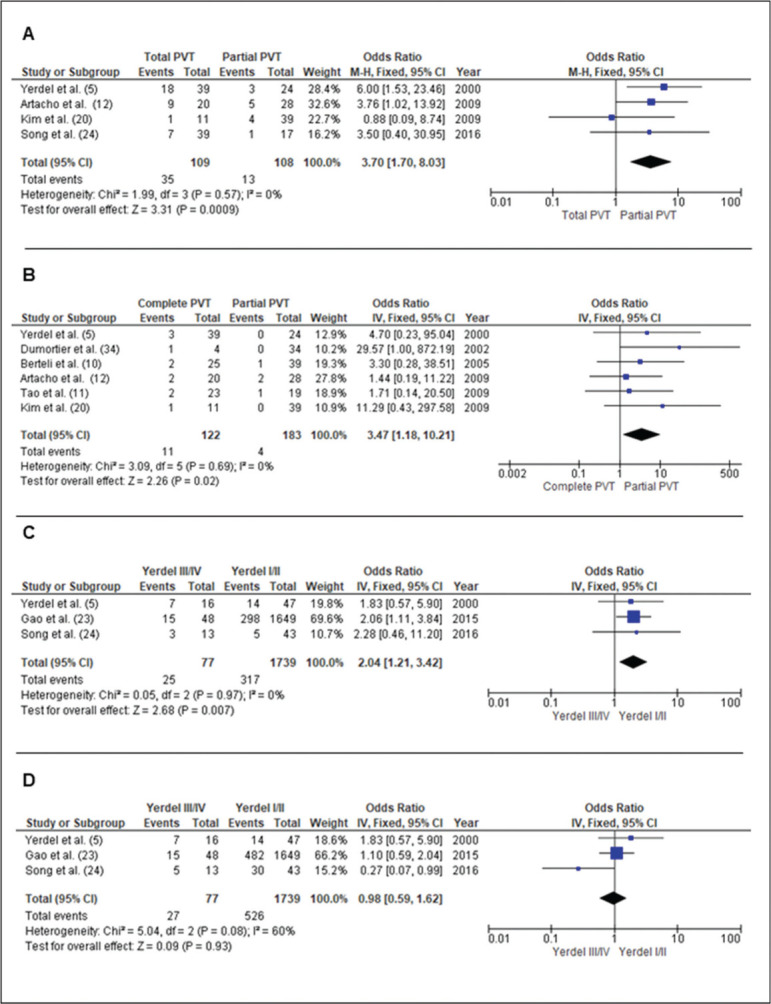
Forrest plots. **A.** Partial *vs* Total PVT (1-year survival). **B**. Rethrombosis **C.** Yerdel classification-1-year Patients Survival. **D.** Yerdel classification-5-year Patient Survival.

Data from four studies (5,12,20,24) evaluated 109 patients with total PVT and 108 patients with partial PVT. The OR was 3.70 [1.70-8.03], *p*=0.0009 (Figure 5A).

#### Rethrombosis after LT

Data from six studies (5,10-12,20,34) evaluated 122 patients with total PVT and 183 patients with partial PVT. The OR for was 3.47 [1.18-10.21], *p*=0.02 (Figure 5B).

#### Patient Survival according to Yerdel classification

Data from three studies (5,23,24) evaluated 77 patients with PVT Yerdel III/IV and 1739 patients with PVT Yerdel I/II. The OR for 1-year and 5-year survival was 2.04 [1.21-3.42], *p*=0.007, and 0.98 [0.59-1.62], *p*=0.93, respectively (Figures 5C and 5D).

## DISCUSSION

PVT may either be a cause or consequence of cirrhosis decompensation. It is asymptomatic in 50% of cirrhotic patients; however, it may lead to severe complications if symptomatic. Early identification and treatment may lead to a better prognosis of these complex patients. The present systematic review and meta-analysis demonstrated that LT in patients with non-tumoral PVT is associated with higher surgery time, higher intraoperative transfusions, and worse 1- and 5-year patient survival. Occlusive PVT, rethrombosis, and Yerdel III/IV also present worse prognosis.

Gao et al. (23), retrospectively analyzing a large casuistic of more than 1800 PVT patients, showed that the overall surgery time of LT was longer in cases with PVT compared to those without it (23). Our meta-analysis, with more than 2,126 PVT patients, confirmed these results.

PVT is considered a very unfavorable prognostic marker in advanced liver disease. Extensive PVT is positively associated with higher mortality during LT (30). One study particularly found that patients with PVT undergoing LT had longer operation time, increased need for transfusions, and lower survival rate than those without PVT (35). Another study revealed similar results, showing that patients with PVT had higher post-transplant mortality than those without PVT. Interestingly, PVT was not associated with increased mortality among patients on the transplant waiting list (15).

Other retrospective studies demonstrated higher intraoperative use of FFP and RBC in PVT cases (10,12,22,29,31). Similar results were found in this meta-analysis. Intraoperative RBC transfusion was evaluated in a total of 4,551 patients (499 with PVT and 4,052 patients without PVT), and a mean difference was 0.80 packs [0.61-0.90] (*p*<0.0001) was found. Furthermore, intraoperative transfusion of FFP (*p*=0.01) and platelets (*p*=0.03) were also higher, comparing 111 patients with PVT to 559 without PVT.

Another meta-analysis by Zanetto et al. (37) identified lower 1-year survival and higher 30 days postoperative mortality in patients with PVT undergoing LT. The survival in partial PVT was better than in the total PVT group (37). We also found lower 1- and 5-year patient survival in the PVT group and lower survival for patients with total occlusive PVT.

Different preoperative strategies have been reported to treat PVT, including anticoagulants and interventional radiology techniques, which may reduce intraoperative technical difficulties and improve outcomes (38-40) and portal vein thrombosis in the living donor (41). In our meta-analysis, thrombectomy with primary anastomosis was associated with better outcomes. Total occlusive PVT presented higher mortality and rethrombosis rates. Accordingly, PVT Yerdel III/IV classification demonstrated worse 1-year and 5-year patient survival.

One limitation of this study is that we found only nonrandomized clinical trials and comparative studies, both prospective and retrospective (Table 1). Therefore, more randomized controlled trials are needed to define more accurate results and improve treatments. Our study shows that proper management of PVT in patients undergoing LT may have real benefit to the clinical practice. Fortunately, some clinical trials are registered in the US National Library of Medicine (https://clinicaltrials.gov). Moreover, we also found in the PROSPERO platform search one ongoing review registered in September 2019 - “Systematic review of surgical techniques for managing porto-mesenteric vein thrombosis in liver transplantation” (PROSPERO Number: CDR42019129755).

## CONCLUSION

In conclusion, LT in patients with non-tumoral PVT demands more surgical time, needs more intraoperative transfusion, and presents worse 1- and 5-year patient survival. In addition to this, total occlusive PVT presents higher mortality and rethrombosis rates, and PVT Yerdel III/IV classification is associated with worse survival. When feasible, thrombectomy with primary anastomosis is associated with better outcomes.

## AUTHOR CONTRIBUTIONS

All authors have approved the final draft of the manuscript submitted. Nacif LS was responsible for the study conception and design, data collection, analysis and interpretation, manuscript writing, and literature search. Zanini LY, Waisberg DR, Pinheiro RS were responsible for the study conception and design, data collection, analysis and interpretation, manuscript critical review. Rocha-Santos V was responsible for the study conception, interpretation and critical revision. Andraus W, Carrilho FJ and Carneiro-D’Albuquerque L were responsible for the study conception, interpretation, and critical review.

## Figures and Tables

**Table 1:** New Castle-Ottawa Scale (NOS) qualification the studies for non-tumoral portal vein thrombosis (PVT) in liver transplantation.

	Selection	Comparability	Outcome	NOS Score
Nonrandomized clinical trial				
Gayovsky et al. (30)	+++	+	++	NOS: 6
Seu et al. (31)	+++	+	+++	NOS: 7
Lerut et al. (32)	++++	+	+++	NOS: 8
Karatzas et al. (33)	+++	+	+++	NOS: 7
Yerdel et al. (5)	++++	+	+++	NOS: 8
Dumortier et al. (34)	+++	+	+++	NOS: 7
Loinanz et al. (27)	+++	+	+++	NOS: 7
Molmenti et al. (29)	+++	+	++++	NOS: 8
Berteli et al. (10)	+++	+	+++	NOS: 7
Gimeno et al. (18)	+++	+	++	NOS: 6
Lendoire et al. (35)	+++	+	++	NOS: 6
Arcadipane et al. (13)	++++	+	+++	NOS: 8
Cho et al. (26)	+++	+	++	NOS: 6
Lladó et al. (21)	++++	+	+++	NOS: 8
Suarez Artacho et al. (12)	+++	+	+++	NOS: 7
Kim et al. (20)	+++	+	+++	NOS: 7
Pan et al. (25)	+++	+	+++	NOS: 7
Tao et al. (11)	++++	+	+++	NOS: 8
Englesbe et al. (15)	++++	+	+++	NOS: 8
Wu et al. (14)	+++	+	+++	NOS: 7
Bhangui et al. (28)	+++	+	++	NOS: 6
Ravaioli et al. (16)	++++	+	+++	NOS: 8
D’Amico et al. (22)	++++	+	+++	NOS: 8
Hibi et al. (19)	++++	+	+++	NOS: 8
Gao et al. (23)	+++	+	++	NOS: 6
Song et al. (24)	+++	+	+++	NOS: 7
Hernandez-Conde et al. (17)	+++	+	+++	NOS: 7

**Note:** NOS, New Castle-Ottawa Score.

**Table 2:** Overall demographics data of the study population of each selected study for non-tumoral portal vein thrombosis (PVT) in liver transplantation.

Article	Baseline data	PVT Classification	PVT - Surgery Technique	Outcome
Gayovsky et al. (30) (n=23) DDLT	Mean age 46 years; 23 males; mean CTP score 12	Yerdel I (n=6) Yerdel III (n=13) Yerdel III (n=13)	6 Thrombectomy 4 Thrombectomy 1 Thrombectomy 1 Graft interposition 11 Jumpgraft SMV	- Worst graft survival (no-PVT group 86% *vs* PVT group 65%, 1 year; p=0.03) - Higher operative blood loss
Seu et al. (31) (n=70) DDLT	48 males and 22 female; 26 (37%) patients with hepatitis cirrhosis	-	61 Thrombectomy 1 Graft interposition 2 Graft interposition coronary vein6 Jumpgraft SMV	-Worst survival -Higher blood transfusion -Higher retransplant rates
Lerut et al. (32 (n=32) DDLT	No demographic data	-	22 Thrombectomy 6 SMV implantation 1 T-T anastomosis with donor PV and SMV receptor 2 Confluence dissection 1 Collateral anastomosis	2 patients underwent thrombectomy and 1 patient underwent confluence dissection died with less of 3 months after LT
Karatzas et al. (33) (n=26) DDLT	No demographic data	-	14 Thrombectomy 8 Jumpgraft SMV 4 CPH	-None interposition failed -Patients with PVT were worst
Yerdel et al. (5) (n=63) DDLT	11% of men presented PVT and 6% of women.High incidence in PVT in patients CTP grade C.	Yerdel I (n=24)Yerdel II (n=23)	4 None 17 low dissection with anastomosis to segment proximal to the thrombus3 Thrombectomy 3 Anastomosis to segment proximal to the thrombus 20 Thrombectomy	-None rethrombosis -Results as well no-PVT -2 patients with rethrombosis. -Higher mortality
		Yerdel III (n=6)	5 Jumpgraft SMV 1 Anastomosis T-T donor PV directly to the receptor PV	-None rethrombosis -Higher mortality
		Yerdel IV (n=10)	2 Thrombectomy 7 Collateral anastomosis 1 Graft interposition collateral	-1 rethrombosis. -Higher mortality (50% overall mortality rate)
Dumortier et al. (34)(n=38) DDLT	No demoghaphic data	Partial PVT (n=34) Total PVT (n=4)	34 Thrombectomy 4 Thrombectomy	-1 patient had recurrence of thrombosis 6 hours after LT (total PVT) -10 patients died after LT with median time 10 months (range 0 - 72 months)
Loinaz et al. (27) (n=76) DDLT	Mean age 45.7±13.7 years; 21 male and 6 female Mean age 48.4±11.3 years; 36 male and 13 female	LT before 1993(group A) (n=27) LT since 1993(group B) (n=49)	75 Thrombectomy 1 Jumpgraft SMV	-Retransplant higher than group B -5 rethrombosis (4 before 3 months) -None rethrombosis -Better survival after LT in comparison of group A
Molmenti et al. (29) (n=85) DDLT	Mean age 53.0±9.1 years ; 54 male and 31 female	-	85 Thrombectomy	-Results as well no -PVT
Berterlli et al. (10) (n=64) DDLT	The only preoperative parameter associated with PVT was CTP grade C(p < 0.005).	Partial PVT (n=39) Complete PVT(n=25)	38 Thrombectomy 1 Jumpgraft SMV 18 Thrombectomy 6 Jumpgraft SMV 1 CPH	-1 patient that underwent thrombectomy had recurrence of PVT. -2 patient underwent thrombectomy had recurrence of PVT.
Gimeno et al. (18) (n=83) DDLT	Mean age 50.61±9.22; 62 men and 21 female. The main LT indication was alcohol cirrhosis	**-**	-	-Higher recurrence of PVT
Lendoire et al. (35) (n=26) DDLT	Median age 40 years (range 17-61); 14 male and 12 female. Preoperative PVT diagnosis In 9 patients. CTP grade C in 61.5%	Yerdel I (n=13) Yerdel II (n=5) Yerdel III (n=5) Yerdel IV (n=3)	13 Thrombectomy 5 Thrombectomy 5 Jumpgraft VMS 3 Thrombectomy	-Better survival rates -2 retrasplantation. -2 retrasplantation. -1 retrasplantation. -2 patients were alive at 68 and 128 months post-transplant, respectively
Arcadipane et al. (13) (n=33) DDLT	Mean age 53.6 years; 20 male and 13 female; mean MELD 21.4	**-**	**-**	-3 patients had recurrence of PVT until 6 months after LT
Cho et al. (26) (n=22) LDLT	Mean age 48.9±10.27; 15 men and 7 women; mean MELD 21.2±7.0	Yerdel I (n=9) Yerdel II (n=6) Yerdel III (n=2)	22 Thrombectomy	Rethrombosis rate was higher in the group with PVT+SMV proximal thrombosis (28.6%), with higher mortality
Lladó et al. (21) (n=42)	Mean age 55.6±7.9;33 men and 9 female; CTP grade A 6 patients, B grade 30 and 6 grade C. The main etiology was alcohol cirrhosis	Yerdel I (n=18) Yerdel II (n=16) Yerdel III (n=6) Yerdel IV (n=2)	12 T-T anastomosis 6 Thrombectomy 9 Thrombectomy 4 SMV confluence 2 Collateral anastomosis1 Graft interposition1 Thrombectomy 1 SMV confluence 2 Collateral anastomosis 2 Jumpgraft SMV2 Collateral anastomosis 1 CPH	None complication 2 patients required stent, due to PV rethrombosis. 1 patient with SMV confluence anastomosis required postoperative stent, due to PV stenosis. 1 patient with collateral anastomosis evaluated with PV stenosis and required stent. The patient underwent caval transposition died due to liver failure.
Artacho et al. (12) (n=48) DDLT	Mean age 53 years; 37 men and 11 female; 38 intraoperative PVT	Partial PVT (n=28)	26 Thrombectomy 1 Jumpgraft SMV 1 CPH	-2 recurrence of PVT -Mortality rates similar with no -PVT
		Complete PVT (n=20 )	10 Thrombectomy 3 Jumpgraft SMV 3 CPH 3 Collateral anastomosis 1 RPA	-2 recurrence of PVT -PVT show higher mortality
Kim et al. (20) (n=50) LDLT	Mean age 52.3±6.7; 27 male and 12 female; CTP score 9.8±1.9; MELD score 16.7±8.5	Partial PVT (n=39)	38 Thrombectomy 1 Graft interposition	-None PVT recurrence -The survival didn’t differed significantly
	Mean age 51.7±7.1; 10 male and 1 female; CTP score 9.6±1.6; MELD score 17.6±6.8	Complete PVT (n=11)	6 Thrombectomy 2 Jumpgraft to SMV 3 Jumpgraft to coronary vein	-1 PV obstruction after LT -The survival did not differed significantly too.
Pan et al. (25) (n=252) DDLT	Sex and previous splenectomy increased the risk of PVT	Yerdel I (n=104) Yerdel II (n=114) Yerdel III (n=29)	104 Thrombectomy 114 Thrombectomy 23 Thrombectomy 4 Jumpgraft to SMV 1 CPH 1 RPA	-Higher blood transfusion in comparison with no-PVT LT -1-year survival wasn’t significantly differed -1 patient Yerdel IV underwent to RPA died due to hepatic failure
		Yerdel IV (n=6)	3 Jumpgraft to SMV 1 CPH 2 RPA	
Tao et al. (11) (n=42) DDLT	Previous treatment of PVT and increasing age are related with higher risk of PVT	Yerdel I (n=19) Yerdel II (n=14) Yerdel III (n=7) Yerdel IV (n=2)	T-T anastomosis with/without thrombectomy 2 Jumpgraft to SMV 2 Donor PV longer anastomosis with recipient PV 3 Anastomosis with coronary vein/splenic vein/umbilical vein 2 CPH	-1 PV rethrombosis -2 patients had postoperative renal failure and 3 LT had graft rejection -2 patients had postoperative renal failure -4 patients presented graft rejection -1 PV rethrombosis -2 patients had postoperative renal failure -2 patients had graft rejection -1 PV rethrombosis
Englesbe et al. (15) (n=897) DDLT	Mean age 54.0±9; MELD score 21.8±8.7	-	-	PVT indicate higher postoperative mortality in LT
Wu et al. (14) (n=24) DDLT (n=6) LDLT (n=18)	Mean age 52.0± 10.0;19 male and 5 female;main etiology was HBV;17 patients had MELD score < 20 and 1 with >40	Yerdel I (n=7) Yerdel II (n=11) Yerdel III (n=6)	13 Thrombectomy 3 Graft interposition 1 Anastomosis to coronary vein 4 Jumpgraft to SMV 3 Jumpgraft to coronary vein	-7 patients died 3 months after LT (1 patient 7^th^ postoperative day due to PVT recurrence follow by graft failure)
Bhangui et al. (28) (n=20) DDLT (n=26)Domino (n=2) LDLT (n=1) Split (n=1)	Mean age 48±12; 14 male and 6 female; Most common etiology was hepatitis C cirrhosis (n=6)	Yerdel IV (n=20)	3 CPH 17 RPA	-1 patient RPA died 3 months after LT, due to a ruptured cerebral vascular malformation (In this case was not evidenced complication related of LT and RPA)
Ravaioli et al. (16) (n=91) DDLT	Mean age 53±9;male 66 and female 25; main etiology was VHC cirrhosis; CTP 1 grade A, 16 grade B and 55 grade C	Partial PVT (n=50) Total PVT (n=41)	50 Thrombectomy 26 Thrombectomy 6 Jumpgraft of SMV 6 CPH 3 Anastomosis with collateral	-Higher recurrence of PVT -Survival was not significantly different among the groups
D’Amico et al. (22) (n=51) DDLT	Mean age 57.5±7.2;male 41 and female 10; main etiology hepatitis cirrhosis; MELD score 18.5±7.1; BMI 24.9±3.2	Partial PVT (n=44) Total PVT (n=7)	39 Thrombectomy 7 Anastomoses at confluence of the SMV 1 T-T anastomosis 4 Extra-anatomic jumpgraft SMV	- PVT increase risk of PVT recurrence and PNF, but didn’t have impact in overall mortality
Hibi et al. (19) (n=174) DDLT	Median age 55 (49-62); 104 male and 45 female; MELD score 20 (16-24); the main etiology was VHC with 71 LT	Physiological reconstruction (n=149)	123 T-T anastomosis 16 Graft Interposition 10 Jumpgraft SMV	-Higher recurrence of PVT
	Median age 54 (47-60);16 male and 9 female; MELD score 18 (15-26); the main etiologies were VHC with 7 VHC and 7 cryptogenic cirrhosis	N-physiological reconstruction (n =25)	6 RPA18 CPH1 Portal vein arterialization	-Longest hospital stay -High in-hospital mortality
Gao et al. (23) (n=1810) DDLT	Mean age 49.81±9.40; 1521 male and 289 female; MELD score 16.85±8.16; the main etiology was VHB	Yerdel I and II (n=1649) Yerdel III (n=26) Yerdel IV (n=22)	1649 T-T anastomosis 19 Jumpgraft SMV 8 Anastomosis T-T portal- SMV 9 CPH 13 Collateral anastomosis	PVT increase the surgical difficult, however didn’t increase mortality rate
Song et al. (24) (n=56) LDLT	Mean age 51.1±8.7;15 male and 2 female; MELD score 17.1±7.3; CTP score 9.5±2.4	Yerdel I (n=17)	17 Thrombectomy	-2 patients with graft rejection -The survival didn’t differed of no -PVT group
	Mean age 51.2±8.3;22 male and 4 female; MELD score 19.0±7.4; CTP score 9.7±1.8	Yerdel II (n=26)	26 Thrombectomy	-2 patients with graft rejection -The survival didn’t differed of no -PVT group
	Mean age 52.1±10.9;9 male and 2 female; MELD score 20.9±9.2; CTP score 11.0±2.0	Yerdel III (n=11)	11 Thrombectomy	-The survival didn’t differed of no -PVT group
	Mean age 51.5±3.5;2 male;MELD score 22.0±15.5; CTP score 9.5±2.1	Yerdel IV (n=2)	1 Thrombectomy1 Jumpgraft SMV	-The 2 patients died until 1 year after LT
Hernandez-Conde et al. (17) 2016 (n=18) DDLT	Mean age 53±5;14 male;MELD score 16.5±4.7 22.0±15.5; CTP 7 grade A, 8 grade B; 2 grade C; Main etiology was VHC with 9 patients	Yerdel I (n=12Y )erdel II (n=3) Yerdel III (n=3)	18 Thrombectomy	The 30-day mortality was higher in PVT patients

**Note:** LT, Liver Transplantation; PVT, portal vein thrombosis; DDLT, deceased donor liver transplantation; LDLT, living donor liver transplantation; MELD, Model for End-stage Liver Disease; CTP, Child-Turcotte-Pugh; SMV, superior mesenteric vein; CPH, cavo-portal hemitransposition; RPA, renal-portal anastomosis; T-T, termino-terminal.
